# BAFF is a marker of hypogammaglobulinemia, neuroaxonal damage and inflammation in multiple sclerosis patients on ocrelizumab

**DOI:** 10.1186/s12974-025-03632-y

**Published:** 2025-11-28

**Authors:** Anastasia Chumakova, Lauren McKay, Victoria Fleming, Michael Demetriou, Michael Sy

**Affiliations:** Department of Neurology, University of California, Irvine, CA USA

## Abstract

**Background and objectives:**

Serum biomarker testing for multiple sclerosis has been increasing in popularity in research and clinical practice. Little evidence is available on influences of disease modifying therapy on serum biomarker levels. Interpretation of clinically available serum biomarkers in the context of each individual patient poses a greater challenge in this context. This study focuses on correlations between clinical variables and unique profile of serum biomarkers in the context of anti-CD20 treatment by ocrelizumab.

**Methods:**

A cohort of multiple sclerosis patients without relapse in the last 12 months and the following 3 months who received serum biomarker testing with the Octave MSDA (Multiple Sclerosis Disease Activity) panel of 18 biomarkers between June 2023 and June 2024 was identified at the UCI Multiple Sclerosis Center. Clinical data was collected retrospectively. Data preparation, analysis and visualization were performed using R.

**Results:**

A total of 118 MS patients without recent acute inflammatory activity were included (63 untreated and 55 on ocrelizumab). Longitudinal immunoglobulin data were available for 48 patients receiving ocrelizumab. Age-adjusted analyses revealed significantly elevated B-cell activating factor (BAFF) levels in the ocrelizumab group. In these patients, BAFF correlated inversely with IgG and IgA—but not IgM—levels. IgG declined over time in patients treated with ocrelizumab, with higher BAFF levels predicting lower IgG and IgA independent of treatment duration. Patients with elevated BAFF exhibited both lower baseline IgG and a more rapid IgG decline compared to those with lower BAFF. Elevated BAFF also correlated positively with markers of neuroaxonal injury, including neurofilament light chain (sNfL) and glial fibrillary acidic protein (GFAP), Myelin oligodendrocyte glycoprotein (MOG), as well as with multiple pro-inflammatory biomarkers such as osteopontin (OPN), CXCL9, CXCL13, CCL20, TRAIL-R1, and CDCP1.

**Discussion:**

This study provides insight into unique biomarker profile in patients on ocrelizumab. Increased BAFF was associated with lower IgG and IgA levels, biomarkers of neuroaxonal damage and inflammation in MS patients without recent acute inflammatory activity on ocrelizumab.

**Supplementary Information:**

The online version contains supplementary material available at 10.1186/s12974-025-03632-y.

## Introduction

Serum biomarkers are increasingly integrated into both research and clinical management of multiple sclerosis (MS), offering the potential to noninvasively monitor disease activity, treatment response, and progression risk [1]. Among the platforms developed to aid in clinical decision-making, the Octave Multiple Sclerosis Disease Activity (MSDA) panel combines 18 serum biomarkers into a composite score intended to reflect current inflammatory activity and predict future relapse risk [2]. However, the interpretation of serum biomarker data remains complex, particularly in the context of disease-modifying therapy (DMT).

Anti-CD20 therapies such as ocrelizumab are among the most effective treatments for relapsing and progressive forms of MS, producing robust suppression of inflammatory disease activity and MRI lesion accrual [3, 4]. Ocrelizumab functions by depleting circulating CD20 + B cells, leading in turn to reduction in Th effector cell activation [5]. However, the specific impact of anti-CD20 therapies on serum biomarker profiles—particularly those included in the Octave MSDA panel—remains poorly characterized.

The clinical utility of serum biomarkers depends not only on their sensitivity to disease activity but also on an understanding of how they are modulated by treatment. In this study, we aimed to elucidate how treatment-related changes may influence biomarker levels independently of disease activity. Using the Octave MSDA panel and longitudinal clinical data, we sought to identify how treatment status and immunologic variables influence clinical serum biomarker interpretability in MS patients without recent acute inflammatory activity receiving ocrelizumab.

## Methods

### Study design and patient selection

We conducted a retrospective analysis of all patients who underwent Octave MSDA serum testing at the University of California, Irvine between June 2023 and June 2024. Cross-sectional data corresponding to the time of the Octave MSDA panel collection were initially extracted. For a subset of patients, longitudinal retrospective data on immunoglobulin levels and infection rates were also collected.

The study was approved by the institutional review board (UCI IRB #5318). The initial dataset included 392 Octave MSDA tests. For patients with multiple tests during the study period, only the earliest available biomarker panel was included. A total of 294 unique patients were retained for further analysis.

### Data collection

Demographic and clinical data collected included age, sex, year of diagnosis, year of symptom onset, height, weight, new symptoms, clinical and MRI stability, weeks since last disease-modifying therapy (DMT) infusion, prior DMTs, and duration since last DMT switch. EDSS was retrospectively captured by review of the physician’s note closest to the Octave MSDA panel testing. MS disease phenotype was assessed by reviewing physician’s notes for 1 year up to the data of Octave MSDA panel collection. MS disease phenotype was not clearly specified in majority of patients' charts, and was determined retrospectively on chart review. Progressive MS (PMS) was defined as evidence of MS-related objective functional decline over the period of 1 year or as a physician’s indication of progression independent of relapse activity (PIRA) in the note. If no objective evidence of decline or PIRA was noted in physician’s documentation, we categorized the patient as Relapsing MS (RMS). Laboratory variables included immunoglobulin levels and Octave MSDA biomarker values. Immunoglobulin levels were obtained from one of the three clinical laboratories. Normal values for IgG ranged between 586–610 mg/dL and 1602–640 mg/dL and were determined by individual laboratories in accordance with CMS guidelines [6]. Hypogammaglobulinemia was defined as IgG levels below the mean for the lower cut-off of 599 mg/dL. Sample processing of Octave MSDA panel test was performed according to previously published validation procedures [7]. Both Octave MSDA panel and immune globulin levels were typically obtained on the same day (36/55 patients), though in some patients (12/55) the collection was on different days, mean time between collection 28.87 days, median time was 0 days, standard deviation was 113.83 days.

Patients were stratified into two groups based on treatment status at the time of Octave MSDA testing: (1) ocrelizumab-treated and (2) no DMT. The no DMT group comprised patients off all DMTs for at least 3 months and off anti-CD20 therapy for at least 2 years. Ocrelizumab-treated group included patients receiving ocrelizumab for a minimum of 6 months. Additionally, only patients with confirmed inflammatory disease stability—defined as no new MRI lesions and no clinical relapses within 12 months prior to and 3 months following the Octave MSDA test—were included. Longitudinal data on immunoglobulin measurements and documented infections were collected for patients in the ocrelizumab group.

### Statistical analysis

Data processing, statistical analyses, and visualization were performed using R version 4.2.0 (2022–04–22) with the following packages: ggpubr, beeswarm, ggplot2, dplyr, forcats, hrbrthemes, viridis, tibble, janitor, lubridate, tidyverse, finalfit, gridExtra, GGally, reshape, pROC, ROCR, lme4, car, lmerTest, emmeans, rstatix, Hmisc, MatchIt, cobalt.

All continuous data were tested for normality using Shapiro–Wilk test. Comparison of age, EDSS and disease duration between the two groups was performed using T test for normally distributed and continuous data and Mann–Whitney test for non-normally distributed or ordinal data.

To balance the no DMT and ocrelizumab groups we performed nearest-neighbor propensity score matching by age, EDSS and prior DMT category. We used the matched groups to perform comparison of biomarker levels between no DMT and ocrelizumab treatment groups.

Generalized linear models were used to evaluate differences in biomarker levels between no DMT and ocrelizumab groups with age and EDSS as covariates. To facilitate the interpretation of significant interaction effects, we used estimated marginal means (EMMs) to examine group differences. These means were calculated based on a reference grid where continuous covariates were held at their mean values. Pairwise comparisons of the EMMs were conducted to assess differences between biomarker levels. The resulting p-values were adjusted for multiple comparisons using the Bonferroni method.

Correlation coefficients (R) between variables were assessed using Pearson correlation test for normally distributed and continuous data, and using Spearman correlation test for non-normally distributed or ordinal data. Correlations with *R* > 0.3 and *p* < 0.05 were considered statistically significant.

Specificity and sensitivity were calculated based on Area Under the Curve using R.

For multiple correlations between biomarkers and clinical variables false discovery rate was controlled by adjusting p-values using Benjamini & Hochberg method.

Generalized linear models were used to assess for correlations. To account for contribution of age and EDSS we compared the following models using ANOVA with Chi-squared test and AIC, and used the model with best fit (Supplemental Table 1): 1) Y ~ X; 2) Y ~ X + Age; 3) Y ~ X + Age + Age^2; 4) Y ~ X + Age + Age^2 + EDSS; 5) Y ~ X + Age + EDSS; 6) Y ~ X + Age + Phenotype; 7) Y ~ X + Age + Phenotype + EDSS; 8) Y ~ X + Age + Age^2 + Phenotype + EDSS.

For modeling of longitudinal immunoglobulin data, several regression models were compared using ANOVA, including random intercept, random slope, and random quadratic slope mixed effect models. Quadratic models were selected for both IgG and IgA based on the lowest deviance and Akaike Information Criterion (AIC) values (Supplemental Table 1). The effects of BAFF on IgG and IgA were analyzed using quadratic models mixed effect models using BAFF as a continuous fixed effect with Type III analysis of variance to assess statistical significance.

## Results

**Fig. 1:**
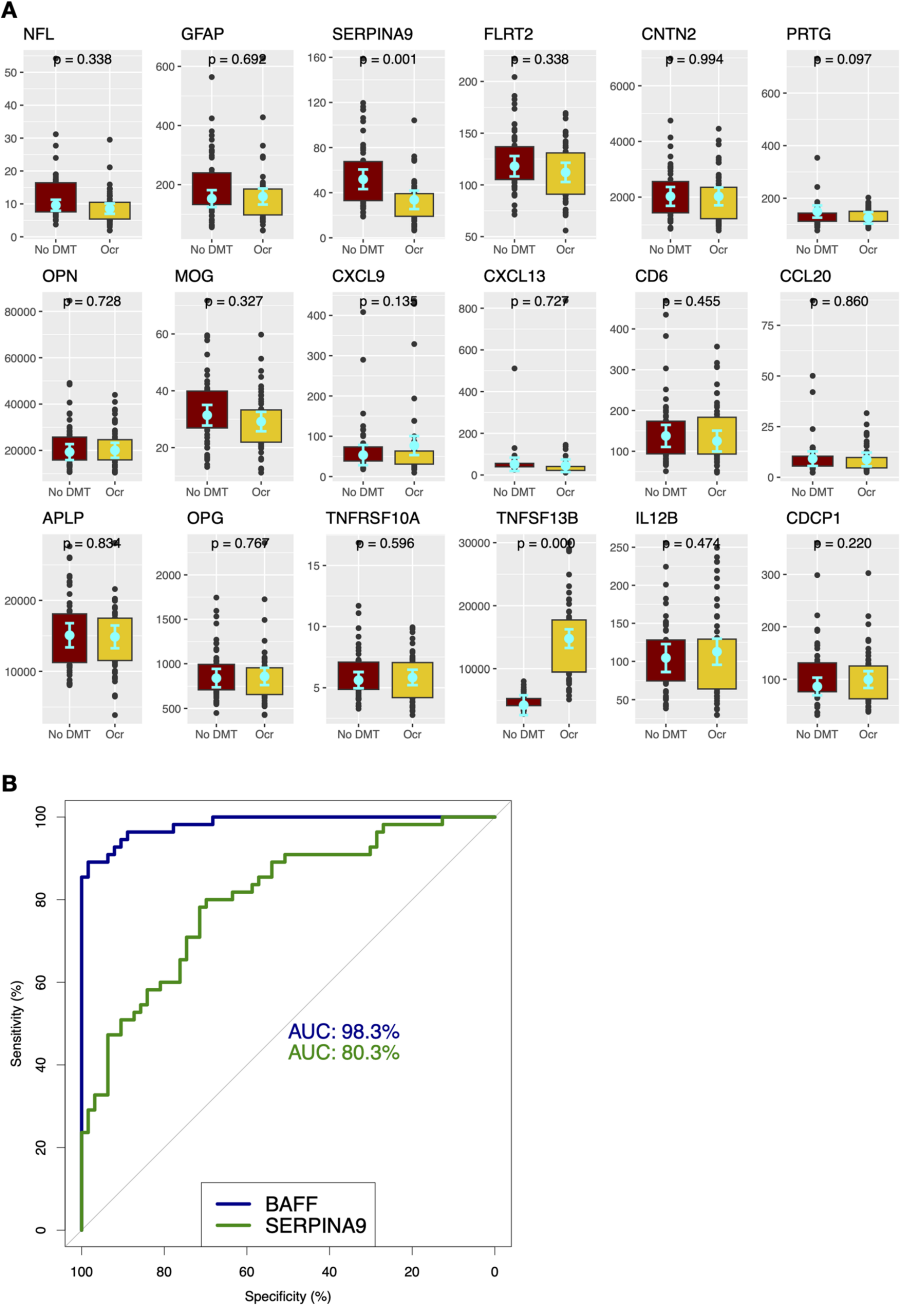
Serum BAFF constitutes a major difference in Octave MSDA biomarker profiles of ocrelizumab patients compared to the no DMT group. **A** Octave MSDA biomarker levels were compared between patients in propensity matched no DMT group and on ocrelizumab (Ocr). Generalized linear mixed model accounting for age and EDSS was used. Estimated marginal means (EMMs) and confidence intervals are presented in cyan color. Adjusted p-values for pairwise EMM comparisons are presented on top of each graph. **B** Receiver operating characteristic (ROC) curves for two serum biomarkers that were found significantly different between no DMT and ocrelizumab groups in A. AUC—area under the curve – were compared between the two biomarkers. BAFF demonstrated highest sensitivity and specificity indicating highest magnitude of effect

A total of 118 MS patients without recent acute inflammatory activity were included in the initial analysis (Table 1), comprising 63 patients not receiving disease-modifying therapy (no DMT) and 55 patients treated with ocrelizumab. Significant differences were observed in age (*p*-value < 0.0001) and disease duration (*p*-value = 0.0001). Patients had similar EDSS levels (*p*-value = 0.477) and a similar proportion of progressive disease phenotype (*p*-value = 0.688) between the two groups. Multiple biomarkers have been shown to correlate with age (Supplementary Fig. 1 A). Accounting for age and EDSS comparisons between the two propensity-matched groups identified two biomarkers that differed significantly: SERPINA9 (*p*-value = 0.0011), and BAFF (*p*-value = < 0.0001) (Fig. 1A). The greatest difference (AUC = 98.3%) was observed in BAFF levels, consistent with the known mechanism of action of ocrelizumab (Fig. 1B).

**Table 1:** Study demographics

		No DMT (*N* = 63)	Ocrelizumab (*N* = 55)	*p* value
Age	Mean (SD)	61.7 (13.2)	47.6 (12.3)	< 0.001
Sex	Female	50 (79.4)	36 (65.5)	0.137
	Male	13 (20.6)	19 (34.5)	
EDSS	Mean (SD)	2.9 (2.4)	2.6 (2.2)	0.447
MS disease phenotype	PMS	13 (20.6)	14 (25.5)	0.688
	RMS	50 (79.5)	41 (74.5)	
Years since diagnosis	Mean (SD)	20.2 (14.4)	11.5 (9.7)	0.001

**Fig. 2:**
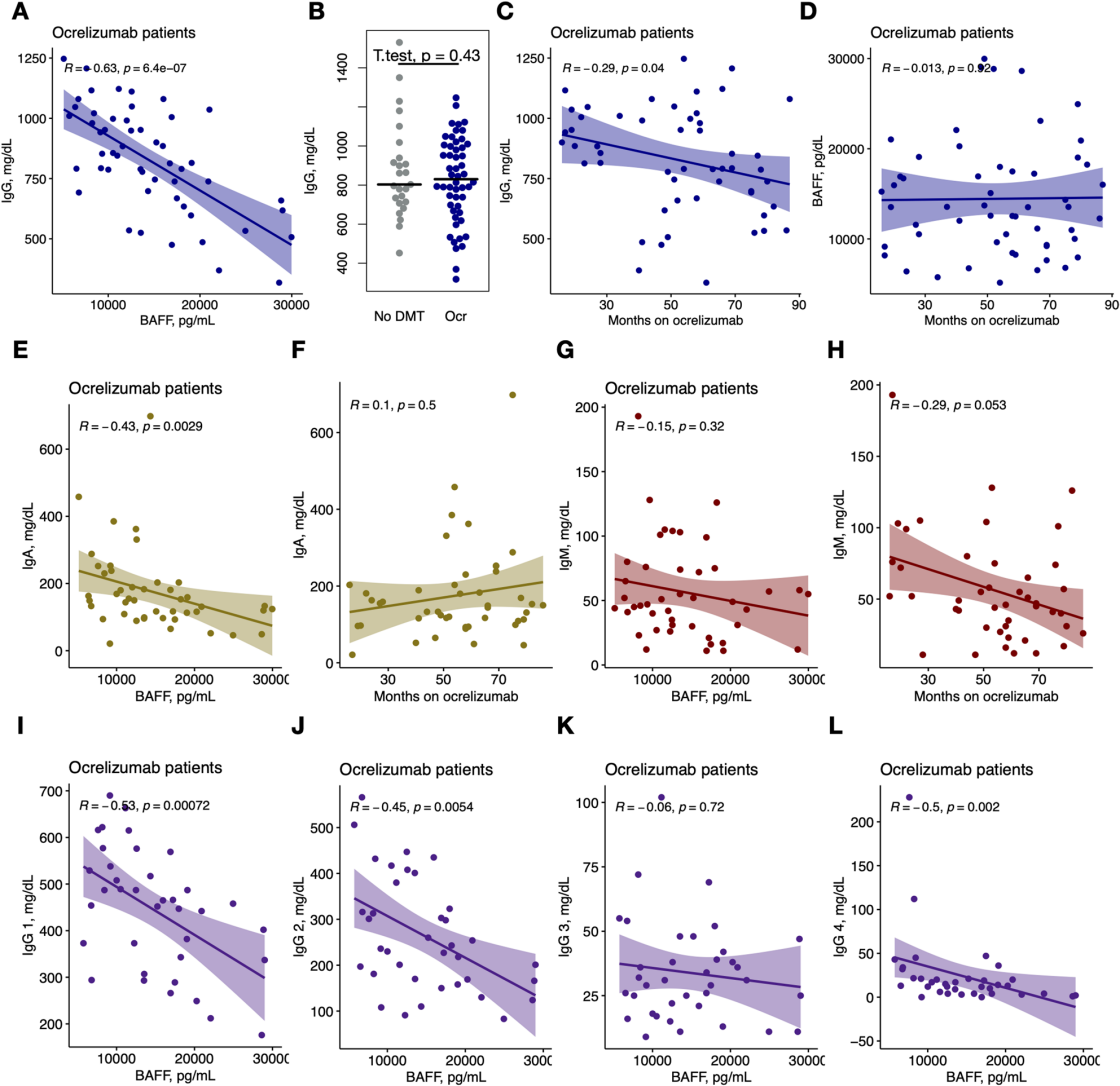
Serum BAFF correlates inversely with immunoglubulin G and A levels. **A** Correlation between serum BAFF and IgG. The best-fitting generalized linear model was found to not include age or EDSS (A, C-L). **B** Comparison of IgG levels between no DMT and ocrelizumab (Ocr) groups. T-test and its p-value shown on the graph. **C** Correlation between months on ocrelizumab and IgG. **D** Correlation between months on ocrelizumab and BAFF. **E** Correlation between serum BAFF and IgA. **F** Correlation between months on ocrelizumab and IgA. **G** Correlation between serum BAFF and IgM. **H** Correlation between months on ocrelizumab and IgM. **I** Correlation between serum BAFF and IgG subclass 1. **J** Correlation between serum BAFF and IgG subclass 2. **K** Correlation between serum BAFF and IgG subclass 3. **L** Correlation between serum BAFF and IgG subclass 4

We next examined associations between clinical variables and biomarker levels, focusing on factors correlations with BAFF (Supplementary Fig. 2). For ocrelizumab patients mean time since last infusion of ocrelizumab to Octave MSDA panel testing was 19.5 weeks (SD 10.05 weeks). Interestingly, we did not observe any influence of the number of weeks since last infusion on any of the biomarker or immune globulin levels (Supplementary Fig. 2). Among patients treated with ocrelizumab, IgG levels demonstrated a strong negative correlation with BAFF (*R* = −0.63, *p* < 0.0001 (Fig. 2A). Total IgG levels were similar (*p*-value = 0.4254) between the ocrelizumab and the no DMT group (Fig. 2B) and showed a trend (*R* = −0.29, p = 0.04) with treatment duration (i.e. months on ocrelizumab prior to sampling; Fig. 2C). However, BAFF levels showed no association (*R* = 0.013, *p* = 0.93) with treatment duration (Fig. 2D).

In addition to total IgG, BAFF levels negative correlated with IgA (*R* = −0.43, *p *= 0.0029), IgG1 (*R* = −0.53, *p* = 0.0007), IgG2 (*R* = −0.45, *p* = 0.0054), and IgG4 (*R* = −0.5, *p* = 0.002) (Fig. 2E, G, I, J), but not with IgM (*R* = −0.15, *p* = 0.32) or IgG3 (*R* = −0.06, *p* = 0.72) (Fig. 2 K, L). Notably, consistent with other studies [8, 9], we found that IgM levels trended down with treatment duration (*R* = −0.29, *p* = 0.053), whereas no such association was observed for IgA (*R* = 0.1, *p* = 0.5) (Fig. 2 F, H).

**Fig. 3:**
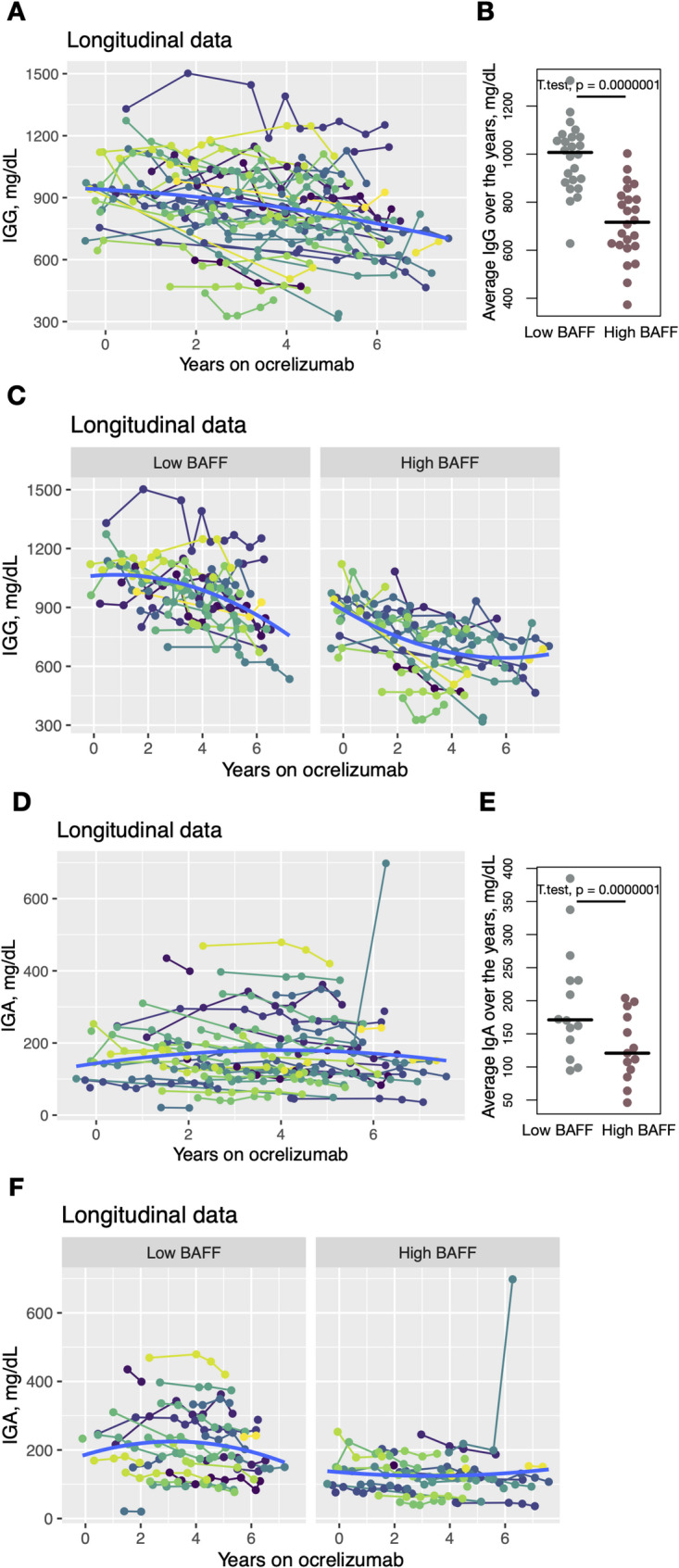
Longitudinal dynamics of IgG levels correspond to serum BAFF level. **A** Longitudinal changes in IgG levels over the years on ocrelizumab (*N* = 48). Quadratic regression was chosen as the best fitting model (blue trend-line). ANOVA was used to evalute for effect of time. **B** Comparison of average IgG over the years on ocrelizumab between patients with below median BAFF (Low BAFF) and above median BAFF (High BAFF) at the time of Octave MSDA panel testing. T-test and it’s *p*-value shown on the graph. **C** Comparison of quadratic regression models between patients with below median BAFF (Low BAFF) and above median BAFF (High BAFF) at the time of Octave MSDA panel testing. ANOVA was used to compare the two regressions. **D** Longitudinal changes in IgA levels over the years on ocrelizumab (*N* = 45). Quadratic regression was chosen as the best fitting model (blue trend-line). ANOVA was used to evalute for effect of time. **E** Comparison of average IgA over the years on ocrelizumab between patients with below median BAFF (Low BAFF) and above median BAFF (High BAFF) at the time of Octave MSDA panel testing. T-test and it’s *p*-value shown on the graph. **F** Comparison of quadratic regression models between patients with below median BAFF (Low BAFF) and above median BAFF (High BAFF) at the time of Octave MSDA panel testing. ANOVA was used to compare the two regressions

Longitudinal IgG levels were available for 48 patients in the ocrelizumab group. Overall, a downward trend in total IgG was observed over time, and quadratic regression analysis confirmed a trend of IgG levels decreasing over time (*p*-value = 0.068) (Fig. 3A). Patients were subsequently stratified into two groups based on the median age-adjusted BAFF level: 27 patients in the low BAFF group and 28 in the high BAFF group. Mean IgG levels across timepoints were significantly higher in the low BAFF group (*p*-value < 0.0001) (Fig. 3B). Furthermore, quadratic regression modeling demonstrated distinct longitudinal IgG dynamics between groups both when using dichotomized BAFF and when BAFF was included as a continuous effect (Fig. 3C, Table 2). Patients with low BAFF had higher baseline IgG levels (*p*-value = 0.00588) and exhibited a delayed decline over the course of treatment. In contrast, the high BAFF group started with significantly lower IgG levels at treatment initiation and experienced a more rapid initial decline over time. A greater proportion of patients in the high BAFF group reached IgG levels < 599 mg/dL during follow-up. In contrast, longitudinal analysis of IgA levels revealed no significant temporal change. Patients with lower BAFF group started with significantly higher IgA level, however there were no statistical differences in group dynamics (Fig. 3 C and D).

**Table 2:** Type III analysis of variance of a mixed effects quadratic regression model of continuous BAFF effect on IgG dynamics. Statistically significant parameters are noted with stars

	NumDF	DenDF	*F* value	Pr(> F)	Signif
BAFF effect on baseline IgG level	1	24.903	9.0748	0.00588	**
BAFF effect on IgG change over time	1	0.468	8.5993	0.006330	**
BAFF effect on rate of IgG change over time	1	36.831	8.5936	0.005767	**

Signif. codes: 0 ‘***’ 0.001 ‘**’ 0.01 ‘*’ 0.05 ‘.’ 0.1 ‘’ 1

**Fig. 4:**
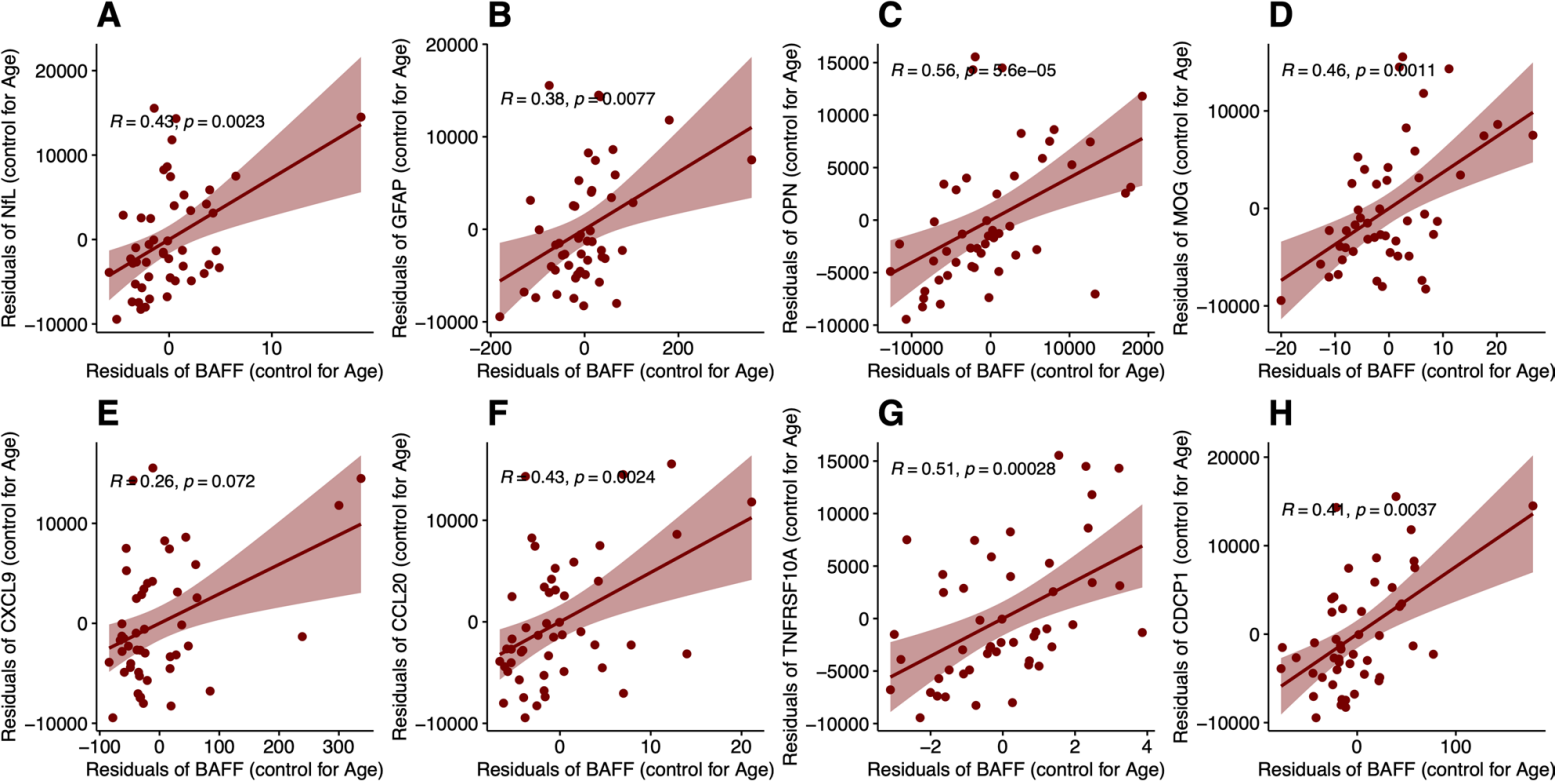
BAFF levels correlate positively with several other biomarkers of neuroaxonal damage and inflammation. **A**-**H** BAFF correlates with other biomarkers of neuroaxonal damage and inflammation in Octave MSDA panel in patients on ocrelizumab. Generalized linear model with age as a covariate was chosen based on best fit. To adjust for the effect of age residuals for BAFF and biomarkers were compared. Correlation coefficients and *p*-values reflect interactions controlled for age

In addition to immunoglobulin levels, several biomarkers associated with neuroaxonal damage and inflammation were positively correlated with BAFF, including neurofilament light chain (NfL) (*R* = 0.43, *p* = 0.0023), glial fibrillary acidic protein (GFAP) (*R* = 0.38, *p* = 0.0077), myelin oligodendrocyte glycoprotein (MOG) (*R* = 0.46, *p* = 0.0011), osteopontin (*R* = 0.56, *p* < 0.0001), CCL20 (*R* = 0.43, *p* = 0.0024), TNFRSF10A (*R* = 0.51, *p* = 0.00028), and CDCP1 (*R* = 0.41, *p* = 0.0037). While on initial analysis CXCL9 showed significant correlation, when accounting for age the correlation did not reach statistical significance (*R* = 0.26, *p* = 0.072). (Fig. 4A–H; Supplementary Fig. 2). In contrast, in the no DMT group, BAFF was only correlated with MOG and IL12B (Supplementary Fig. 3). We did not observe a relationship between BAFF, IgG and IgM levels with infection rates over the previous 2 years in the ocrelizumab group, even when duration of ocrelizumab exposure was included as a covariate (Supplementary Fig. 4).

## Discussion

Anti-CD20 therapies are among the most widely used DMTs in MS due to their robust efficacy in preventing clinical relapses and radiologic activity [3]. The Octave MSDA panel was developed to quantify disease activity using serum biomarkers and support risk stratification in clinical practice [2]. BAFF, a central regulator of B-cell homeostasis, contributes significantly to the MSDA score, likely reflecting both the method of algorithm development and the low inflammatory biomarker profile observed in anti-CD20–treated patients with stable MRI findings [2, 7]. Our findings suggest that lower BAFF levels observed in untreated patients are not indicative of increased disease activity, highlighting the complexity of BAFF biology in MS. Though our cohort did not provide evidence of significant differences between many other biomarker levels besides SERPINA9 and BAFF in ocrelizumab-treated patients compared to controls, larger-scale longitudinal studies are needed to interrogate treatment effect on other Octave MSDA panel biomarkers.

The primary long-term concern with anti-CD20 therapy is infection risk, largely attributed to treatment-induced hypogammaglobulinemia (HypoIgG). HypoIgG has been reported in approximately 10–15% of patients treated with rituximab [10], with similar frequencies noted in ocrelizumab-treated cohorts [11]. BAFF levels are known to rise following B-cell depletion, reflecting homeostatic feedback [12]. Early in the treatment course (within the first year) some studies have described gradual increase of BAFF levels following initiation of rituximab [13]. Interestingly, in our cohort we observed no correlation between BAFF and duration of B-cell depletion over years on the medication. This may be attributed to the lack of early data within the first year after treatment initiation in our cohort. Furthermore, we observed no correlation between weeks since last ocrelizumab infusion and any of the biomarker or clinical data we collected, which may suggest relative stability of the variables over the 6 month period. In our cohort BAFF did not correlate with age or MS disease phenotype, which is consistent with a known link between age and progressive phenotype of MS [14]. Elevated BAFF has been associated with increased infection risk, potentially serving as a biomarker of immunologic imbalance [15]. Our data support a mechanistic link, showing that elevated BAFF is associated with a more pronounced decline in IgG over time, potentially increasing susceptibility to infections. The lab-specific cut-offs for hypoIgG in our study seem to be lower than in other similar studies, which should be taken into account when applying our results to the interpretation of absolute values of IgG levels. Ultimately, a larger scale longitudinal study of both IgG and BAFF dynamics before and after B-cell depletion is needed to elucidate the role of each factor in the development of hypoIgG. In larger studies HypoIgG has been established as a major risk factor for the development of infections [16–18]. These studies have also established a link between time and dose exposure to anti-CD20 treatments and infection risk. Though our study did not observe correlation between IgG and infection risk, this is likely due to a smaller study size, as other studies of similar size have run into the same problem. Interestingly, in our cohort we did not observe a correlation between months on ocrelizumab and infection risk. This is likely underrepresented in our data due to study size and retrospective design. Ultimately, HypoIgG may represent an independent risk factor for increased risk of infections, which makes early prediction of this complication using biomarker testing an potentially important clinical tool.

Interestingly, among immunoglobulin isotypes, BAFF levels correlated with IgG and IgA, but not IgM. While BAFF is critical for the survival and maturation of naive B cells [19], its role in sustaining class-switched memory B cells is less consistent. In some immune-mediated conditions, elevated BAFF levels have been inversely associated with switched memory B cells [20]. This may explain why BAFF correlated with IgG and IgA (products of class-switched cells), but not IgM, which is primarily secreted by short-lived, non-switched B cells [21].

Longitudinal analysis in our cohort demonstrated that BAFF plays a role in the dynamics of IgG decline over the course of anti-CD20 therapy. Importantly, BAFF levels were not correlated with treatment duration, suggesting that inter-individual differences in BAFF response may be intrinsic and potentially detectable early in treatment. Given the strong baseline correlation between BAFF and IgG, pre-treatment IgG levels may serve as a predictive marker of BAFF response phenotype and risk for subsequent HypoIgG. Patients with lower baseline IgG tended to have higher BAFF levels and experienced a more rapid decline in IgG over time. Similar patterns have been observed in common variable immunodeficiency (CVID), where elevated BAFF levels reflect impaired class-switched memory B-cell compartments and may promote naive B-cell survival as a compensatory mechanism [22, 23].

In other autoimmune diseases, elevated BAFF levels are associated with adverse clinical outcomes. In systemic lupus erythematosus (SLE), BAFF blockade with agents such as belimumab has demonstrated significant clinical benefit and is now an approved therapy [24]. In contrast, attempts to target BAFF in MS have yielded mixed results. The pivotal phase II trial of atacicept, an anti-BAFF/APRIL agent, was terminated early due to increased disease activity and relapses in treated patients, highlighting the complexity of BAFF’s role in MS pathogenesis [25, 26]. More recent investigations have continued to explore BAFF biology in this context. Ho et al. (2023) reported heterogeneous BAFF signaling and B-cell reconstitution patterns following anti-CD20 therapy, suggesting that individual variability in BAFF pathways may impact treatment outcomes and immune risk profiles [27]. Conversely, Gupta et al. (2023)provided evidence that elevated BAFF levels post-B-cell depletion might support regulatory B-cell populations and help limit CNS autoimmunity, cautioning against indiscriminate BAFF suppression [28]. A recent study by Wang et al. (2024)demonstrated a potential neuroprotective role of BAFF in murine models and reported elevated BAFF levels in patients receiving anti-CD20 therapies [29]. However, our data revealed that in ocrelizumab treated patients BAFF levels positively correlated with multiple biomarkers of neuroaxonal damage, including NfL and GFAP. Together, these findings underscore the complex and potentially population-dependent role of BAFF in MS and highlight the need for personalized therapeutic strategies when considering B-cell targeted interventions. This study was limited due to small sample size, retrospective design and lack of longitudinal data on BAFF levels before and during ocrelizumab treatment. Further larger-scale prospective studies are warranted to more clearly elucidate the role of BAFF in the complex landscape created by B-cell depletion.

## Supplementary Information

Supplementary Material 1: Supplementary Fig. 1. Correlations between age and Octave MSDA serum biomarkers in patients on no DMT

Supplementary Material 2: Supplementary Fig. 2. Correlations between BAFF, other clinical variables and serum biomarkers in patients on ocrelizumab. *P*-values adjusted for false discovery rate are shown and green indicates statistically significant correlations.

Supplementary Material 3: Supplementary Fig. 3. Correlations between BAFF, other clinical variables and serum biomarkers in patients on no DMT. *P*-values adjusted for false discovery rate are shown and green indicates statistically significant correlations.

Supplementary Material 4: Supplementary Fig. 4. Correlations between infection rates over the past 2 years, BAFF and immuno globulin levels. A Correlation between BAFF and total number of infections in prior 2 years. B Correlation between IgG and total number of infections in prior 2 years. C Comparison of total number of infections in the prior 2 years between below median BAFFand above median BAFFgroups. D Comparison of major infections requiring hospitalization in the prior 2 years between below median BAFFand above median BAFFgroups. E Correlation between IgM levels and major infections requiring hospitalization in the prior 2 years. F Correlation between IgM levels and total number of infections in prior 2 years

Supplementary Material 5: Supplementary Table 1. Analysis of best fit on the models used in the manuscript for data in each figure

## Data Availability

The data supporting the findings of this study are available from the corresponding author upon reasonable request. Deidentified participant data may be shared in accordance with institutional policies and IRB approval. Requests for data access should include a brief research proposal and intended use.
