# Supplementary figures and images for: BAFF is a marker of hypogammaglobulinemia, neuroaxonal damage and inflammation in multiple sclerosis patients on ocrelizumab
Source: J Neuroinflammation. 2025 Nov 28;23:2. doi: 10.1186/s12974-025-03632-y (PMC12765282; doi:10.1186/s12974-025-03632-y)

# Supplement 1

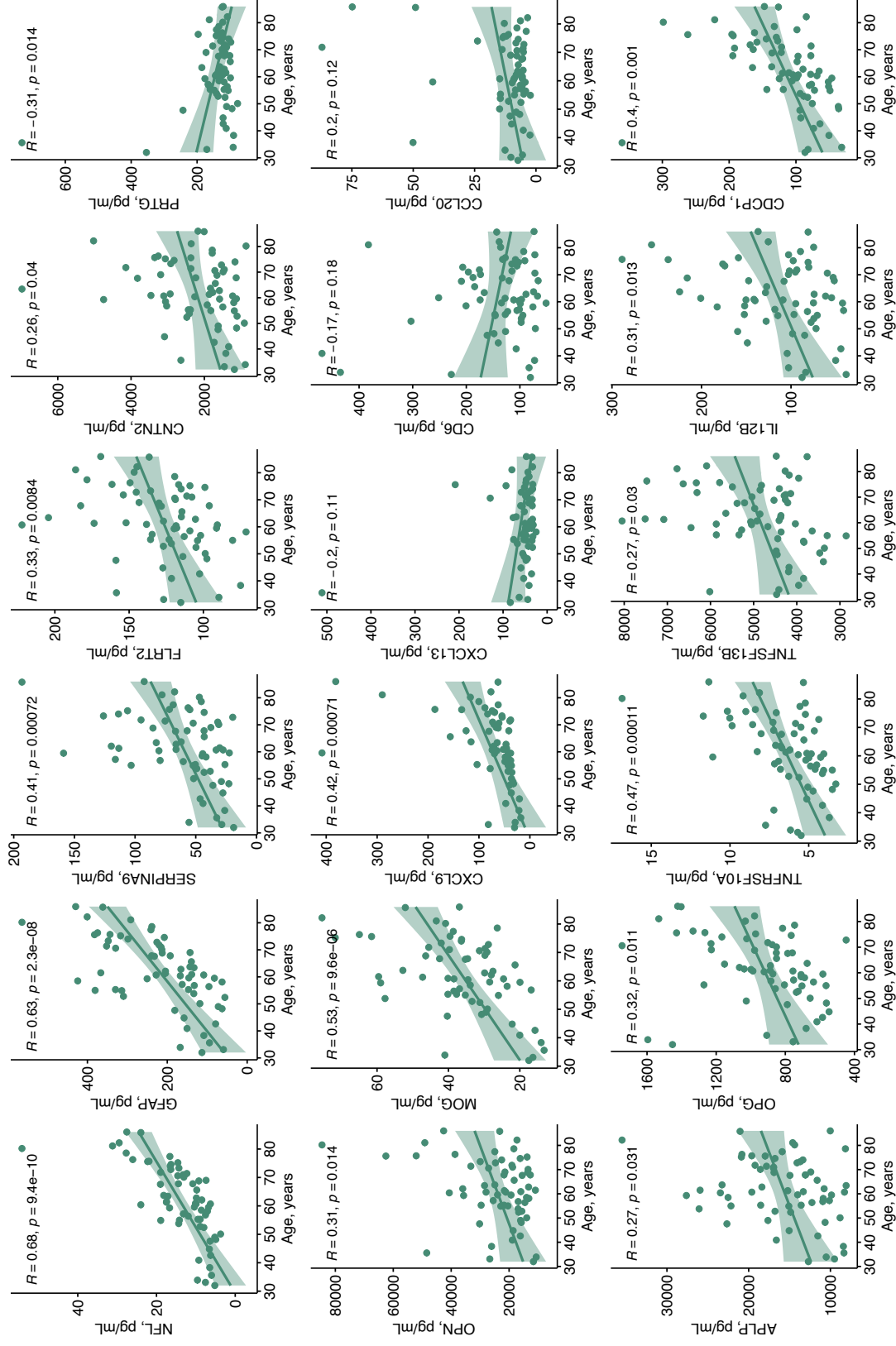

Supplement: Supplementary file 1 — Supplementary Material 1: Supplementary Fig. 1. Correlations between age and Octave MSDA serum biomarkers in patients on no DMT [file 12974_2025_3632_MOESM1_ESM.pdf]

Supplement 4

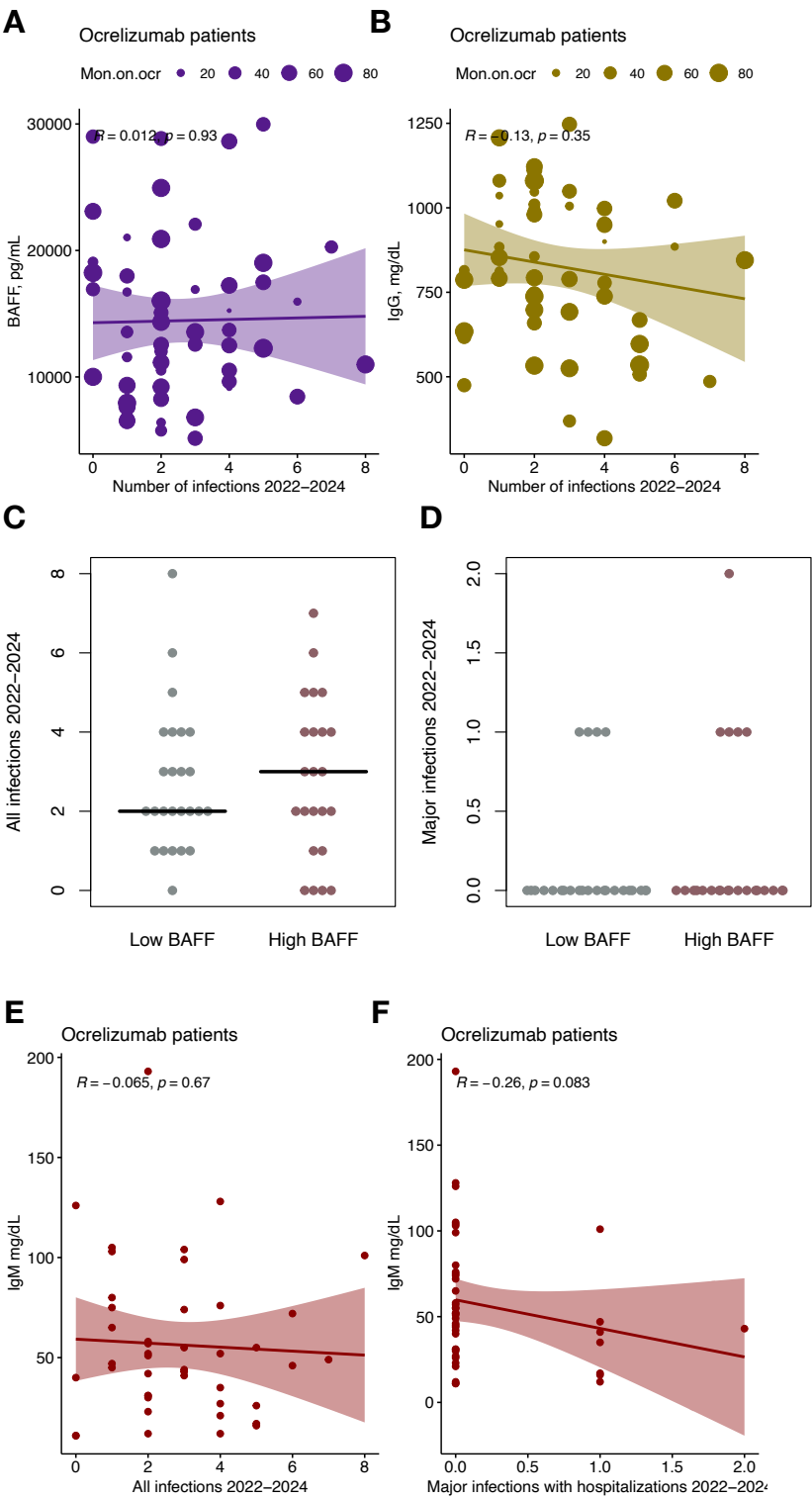

Supplement: Supplementary file 4 — Supplementary Material 4: Supplementary Fig. 4. Correlations between infection rates over the past 2 years, BAFF and immuno globulin levels. A Correlation between BAFF and total number of infections in prior 2 years. B Correlation between IgG and total number of infections in prior 2 years. C Comparison of total number of infections in the prior 2 years between below median BAFFand above median BAFFgroups. D Comparison of major infections requiring hospitalization in the prior 2 years between below median BAFFand above median BAFFgroups. E Correlation between IgM levels and major infections requiring hospitalization in the prior 2 years. F Correlation between IgM levels and total number of infections in prior 2 years [file 12974_2025_3632_MOESM4_ESM.pdf]
