# Supplementary material for: BAFF is a marker of hypogammaglobulinemia, neuroaxonal damage and inflammation in multiple sclerosis patients on ocrelizumab
Source: J Neuroinflammation. 2025 Nov 28;23:2. doi: 10.1186/s12974-025-03632-y (PMC12765282; doi:10.1186/s12974-025-03632-y)
Supplement: Supplementary file 2 — Supplementary Material 2: Supplementary Fig. 2. Correlations between BAFF, other clinical variables and serum biomarkers in patients on ocrelizumab. P-values adjusted for false discovery rate are shown and green indicates statistically significant correlations. [file 12974_2025_3632_MOESM2_ESM.pdf]

Supplement 2

	**BAFF**																													
Height	0.4104	**Height**																												
Weight	0.6439	0.0208	**Weight**																											
Years since diagnosis	0.9781	0.6770	0.9567	**Years since diagnosis**																										
Months on ocrelizumab	0.8886	0.7621	0.9149	0.0507	**Months on ocrelizumab**																									
EDSS	0.8331	0.9307	0.6865	0.0030	0.3257	**EDSS**																								
Weeks since infusion	0.9843	0.6556	0.7703	0.1300	0.3214	0.6838	**Weeks since infusion**																							
IGG	0.0000	0.2175	0.7506	0.2311	0.1455	0.3828	0.6865	**IgG**																						
IGA	0.1150	0.4987	0.3656	0.8331	0.5457	0.5457	0.7506	0.1719	**IgA**																					
IGM	0.5156	0.9154	0.7139	0.2311	0.1455	0.9307	0.9539	0.1953	0.5858	**IgM**																				
IGG1	0.0195	0.1819	0.6431	0.2878	0.4809	1.0000	0.7887	0.0000	0.4180	0.1819	**IgG1**																			
IGG2	0.0368	0.4994	0.8331	0.6865	0.3120	0.8331	0.3165	0.0001	0.6838	0.3656	0.4987	**IgG2**																		
IGG3	0.7033	0.9307	0.5407	0.8767	0.4912	0.8331	0.5794	0.1150	0.9808	0.3656	0.4712	0.1302	**IgG3**																	
IGG4	0.1505	0.1518	0.5720	0.9776	0.8331	0.7923	0.9264	0.0584	0.9149	0.7415	0.3476	0.6803	0.9843	**IgG4**																
NfL	0.0072	0.6717	0.5558	0.6691	0.8331	0.0393	0.5941	0.0584	0.6838	0.7506	0.8331	0.3347	0.6787	0.5705	**NfL**															
GFAP	0.0202	0.2866	0.1073	0.6717	0.3257	0.2346	0.9567	0.0236	0.5794	0.9781	0.3592	0.1486	0.6992	0.4912	0.0030	**GFAP**														
SERPINA9	0.8686	0.9149	0.6865	0.6063	0.9945	0.8907	0.6274	0.8524	0.9149	0.5815	0.4653	0.8413	0.0733	0.8314	0.9307	0.2346	**SERPINA9**													
FLRT2	0.2129	0.7621	0.8935	0.8705	0.8331	0.6838	0.9776	0.4912	0.8935	0.6431	0.9264	0.2552	0.8756	0.4790	0.8849	0.7874	0.4366	**FLRT2**												
CNTN2	0.5815	0.5815	0.9530	0.7506	0.8331	0.7506	0.4991	0.8331	0.3377	0.6431	0.9379	0.7506	0.7232	0.8331	0.8113	0.1444	0.5558	0.3596	**CNTN2**											
PRTG	0.1073	0.6838	0.5558	0.7801	0.6044	0.7923	0.5558	0.4991	0.8331	0.9567	0.4492	0.6865	0.9930	0.2621	0.3214	0.1009	0.6865	0.1789	0.0183	**PRTG**										
OPN	0.0039	0.8849	0.5794	0.5311	0.8200	0.7506	0.7664	0.0874	0.5794	0.6431	0.2878	0.6717	0.4595	0.7621	0.0828	0.1455	0.8366	0.3992	0.5140	0.3411	**OPN**									
MOG	0.0021	0.5457	0.0349	0.9860	0.8210	0.9493	0.7621	0.3592	0.6865	0.5350	0.6691	0.2339	0.8413	0.9843	0.0392	0.0008	0.4595	0.4492	0.0354	0.1240	0.0576	**MOG**								
CXCL9	0.0215	0.8284	0.8085	0.6717	0.8331	0.2488	0.9264	0.1155	0.5705	0.9111	0.5794	0.3828	0.3592	0.6865	0.0001	0.0659	0.6691	0.6677	0.4496	0.5794	0.0634	0.2705	**CXCL9**							
CXCL13	0.1300	0.6865	0.8331	0.1493	0.8935	0.5858	0.5815	0.1455	0.5457	0.5815	0.2945	0.5794	0.6838	0.6865	0.4912	0.0562	0.8331	0.2920	0.4912	0.5941	0.0575	0.5152	0.0007	**CXCL13**						
CD6	0.4458	0.9860	0.9083	0.6717	0.8331	0.5815	0.4987	0.8305	0.6838	0.7317	0.6717	0.5794	0.6556	0.5156	0.9009	0.6838	0.9437	0.1819	0.8200	0.6691	0.7535	0.5675	0.9781	0.8200	**CD6**					
CCL20	0.0119	0.7170	0.8331	0.8147	0.8935	0.8331	0.9930	0.0129	0.7185	0.7801	0.0300	0.3411	0.5152	0.6838	0.2556	0.0562	0.6253	0.7170	0.7506	0.2495	0.0349	0.4458	0.0051	0.0021	0.8331	**CCL20**				
APLP	0.5152	0.0393	0.0021	0.7874	0.9861	0.9607	0.7786	0.9776	0.9567	0.3992	0.7764	0.7745	0.6431	0.8016	0.7801	0.3129	0.6717	0.8380	0.1555	0.9607	0.5080	0.0004	0.8767	0.8692	0.8349	0.4366	**APLP**			
OPG	0.5312	0.0634	0.0256	0.9860	0.9111	0.7461	0.5060	0.4580	0.3214	0.8909	0.1150	0.6787	0.9876	0.5323	0.5558	0.2600	0.7086	0.8331	0.9178	0.0659	0.4912	0.0828	0.1023	0.6838	0.9860	0.8331	0.6865	**OPG**		
TNFRSF10A	0.0028	0.2311	0.4790	0.6520	0.8914	0.6770	0.9405	0.0561	0.8331	0.7801	0.1719	0.3992	0.9740	0.4994	0.0119	0.2130	0.8908	0.6865	0.8331	0.2945	0.0431	0.0561	0.0039	0.1150	0.6982	0.1819	0.8331	0.0021	**TNFRSF10A**	
IL.12B	0.6865	0.9945	0.8366	0.8692	0.4912	0.9860	0.7506	0.9592	0.6717	0.6865	0.6152	0.6865	0.9567	0.4991	0.4790	0.8536	0.3406	0.6431	0.9592	0.4912	0.7801	0.6971	0.4912	0.8692	0.7374	0.8366	0.9197	0.4458	0.3214	**IL12B**
CDCP1	0.0016	0.8692	0.9418	0.4580	0.5705	0.1073	0.8767	0.0300	0.5558	0.6152	0.8331	0.0659	0.6865	0.7506	0.0000	0.0024	0.4805	0.8849	0.8692	0.6152	0.1155	0.0659	0.0000	0.1171	0.8016	0.0507	0.8692	0.3656	0.0012	0.2511
