# Supplementary material for: BAFF is a marker of hypogammaglobulinemia, neuroaxonal damage and inflammation in multiple sclerosis patients on ocrelizumab
Source: J Neuroinflammation. 2025 Nov 28;23:2. doi: 10.1186/s12974-025-03632-y (PMC12765282; doi:10.1186/s12974-025-03632-y)
Supplement: Supplementary file 3 — Supplementary Material 3: Supplementary Fig. 3. Correlations between BAFF, other clinical variables and serum biomarkers in patients on no DMT. P-values adjusted for false discovery rate are shown and green indicates statistically significant correlations. [file 12974_2025_3632_MOESM3_ESM.pdf]

Supplement 3

	BAFF																			
**Years since diagnosis**	0.6605	**Years since diagnosis**																		
**EDSS**	0.5038	0.0321	**EDSS**																	
**IgG**	0.2875	0.1050	0.2756	**IgG**																
**NfL**	0.2171	0.4910	0.0001	0.1286	**NfL**															
**GFAP**	0.5265	0.4407	0.0261	0.4921	0.0000	**GFAP**														
**SERPINA9**	0.6649	0.8228	0.6366	0.8673	0.1650	0.1542	**SERPINA9**													
**FLRT2**	0.3690	0.9859	0.5208	0.5115	0.0236	0.1051	0.7395	**FLRT2**												
**CNTN2**	0.2109	0.8839	0.8228	0.1707	0.3088	0.4047	0.5129	0.0013	**CNTN2**											
**PRTG**	0.7744	0.3047	0.8228	0.5379	0.5208	0.5115	0.6146	0.1742	0.5114	**PRTG**										
**OPN**	0.5610	0.9859	0.3041	0.6146	0.0000	0.0017	0.6909	0.0358	0.7261	0.1341	**OPN**									
**MOG**	0.0030	0.9859	0.1225	0.1602	0.0007	0.0001	0.1542	0.1141	0.0221	0.2153	0.5208	**MOG**								
**CXCL9**	0.5208	0.5825	0.1232	0.5545	0.0104	0.1225	0.9769	0.0767	0.9988	0.5709	0.0216	0.4854	**CXCL9**							
**CXCL13**	0.8498	0.6180	0.8228	0.3587	0.7509	0.7744	0.5379	0.4711	0.6909	0.0000	0.0401	0.6571	0.8651	**CXCL13**						
**CD6**	0.6909	0.4619	0.9826	0.5185	0.8383	0.7394	0.2875	0.3730	0.8773	0.5709	0.7235	0.9826	0.7261	0.7135	**CD6**					
**CCL20**	0.8839	0.4653	0.8817	0.1542	0.3797	0.4821	0.1894	0.5379	0.7261	0.7744	0.5115	0.7744	0.0129	0.9769	0.6180	**CCL20**				
**APLP**	0.2705	0.8638	0.6257	0.1542	0.0825	0.0035	0.8540	0.5345	0.0325	0.5453	0.9769	0.0000	0.9826	0.5185	0.8121	0.8651	**APLP**			
**OPG**	0.2548	0.8730	0.4198	0.6909	0.0475	0.0178	0.2042	0.0560	0.5114	0.6146	0.0249	0.0887	0.0443	0.3797	0.5317	0.2875	0.7193	**OPG**		
**TNFRSF10A**	0.2548	0.4921	0.0027	0.9928	0.0000	0.0001	0.5714	0.0475	0.8399	0.8638	0.0000	0.1286	0.0001	0.5208	0.6961	0.1742	0.7915	0.0005	**TNFRSF10A**	
**IL.12B**	0.0141	0.5825	0.7261	0.1542	0.1542	0.1542	0.6146	0.0858	0.4899	0.9826	0.0893	0.0518	0.0852	0.7261	0.6909	0.6961	0.4921	0.1542	0.1209	**IL12B**
**CDCP1**	0.2077	0.78917	0.03251	0.99882	3.53929	0.00370	0.99882	0.00177	0.20766	8.37257	7.28663	0.15418	0.08867	8.18192	0.86377	0.51011	0.85403	0.02035	5.31865	0.05603
